# Pediatric Endotracheal Tube Cuff Management at Altitude: Implications for Aeromedical Retrieval and Other Austere Environments

**DOI:** 10.1111/pan.15123

**Published:** 2025-05-01

**Authors:** Matthew Desmond, Britta S. von Ungern‐Sternberg

**Affiliations:** ^1^ Royal Flying Doctor Service (Queensland Section) Cairns Australia; ^2^ College of Medicine & Dentistry James Cook University Cairns Australia; ^3^ Department of Anaesthesia and Pain Medicine Perth Children's Hospital Nedlands Australia; ^4^ Perioperative Medicine Team, Perioperative Care Program The Kids Research Institute Australia Nedlands Australia; ^5^ Institute for Paediatric Perioperative Excellence The University of Western Australia Perth Australia; ^6^ Division of Emergency Medicine, Anaesthesia and Pain Medicine The University of Western Australia Perth Australia

**Keywords:** aeromedical transport, altitude, cuff pressure, pediatric endotracheal tube

## Abstract

**Background and Objectives:**

Children are sometimes transported via fixed or rotary wing aircraft for medical care. If they are intubated with a cuffed endotracheal tube (ETT), changes in environmental pressure during transport can alter cuff pressure. Cuff management in this setting varies widely by region and by organization. In this historical review, we sought to delineate the evolution of ETT cuff management in children undergoing aeromedical retrieval in order to progress the field toward an optimum strategy in the future.

**Descriptions and Conclusions:**

Problems with extremely high ETT cuff pressures in adults due to altitude gain were identified by the 1970s. During subsequent decades, this topic was the subject of fervent research and device development, with a relative waning in interest more recently. Children, being transported less frequently and almost always with non‐cuffed ETTs, were not included in these research efforts. During a similar epoch, the field of hyperbaric medicine also recognized the issue of ETT cuff pressure changes and almost uniformly changed to cuff insufflation with an incompressible liquid. This was based on cuff pressure measurements and deductive reasoning, rather than on evidence from patient outcome trials. Aeromedical retrieval has not consistently adopted this technique. Further investigation and discussion on an optimum strategy of cuff management in aeromedical transport of children is needed to reach an agreement on best practice.

## 1 Introduction

Pediatric respiratory mucosa is delicate, and the airway is prone to trauma‐related complications. A small change in airway diameter caused by mucosal swelling significantly affects airway resistance in small children. Therefore, endotracheal tube (ETT) cuff pressure is of particular interest. Altitude gain leads to ETT cuff expansion and, therefore, a higher cuff pressure. With descent, cuff pressure lessens, and a cuff leak can result. This pressure vis‐a‐vis aeromedical retrieval has been a concern for decades yet has mainly been studied in adults. In the past, this was due to the standard practice of utilizing only uncuffed tubes in children. Thus, during the first decades of aeromedical retrieval, when clinicians were alerted to this issue by hypobaric scenarios and conducted the first research studies, children were not involved. More recently, the paucity of pediatric research is more likely related to a decrease in interest and awareness, the relatively few flights compared to the adult population, and additional regulatory barriers.

Most institutions now use cuffed ETTs for children, impacting retrieval operations. The benefits of cuffed ETTs, such as improved ventilation, lower ETT exchange rates, and decreased ETT movement, are particularly important during air transport [1]. However, infants and children are more likely to have an inappropriate volume injected in the cuff and are more likely to have airway difficulties due to intraluminal swelling, for example, edema or subglottic stenosis [2]. The tracheal mucosa is vulnerable—not intended to withstand internal pressure—and injury occurs within 15 min when the cuff pressure is too high [3, 4, 5]. In adults, for example, an intracuff pressure above 30 cm H_2_O for 15 min induces histological evidence of tracheal mucosal lesions and impairs mucosal blood flow [5].

Helicopters routinely fly patients at 3000 to 5000 ft above sea level without pressurized cabins, while pressurized airplanes commonly have cabin pressures equal to 8000 ft above sea level. Some retrievals in alpine environments at even higher altitudes can involve multiple in‐flight ascents and descents. These altitude alterations can cause dramatic cuff pressure changes if air is used for insufflation but very little if an incompressible fluid (such as saline or water) is used. However, not all retrieval services use liquid to fill ETT cuffs, including in pediatric scenarios. Here, we review the status of the literature and practice regarding ETT cuff management in pediatric aeromedical rescue and retrieval.

## 2 Evaluating Cuffs and the Tracheal Mucosa

Cuff pressure in high‐volume, low‐pressure ETTs is essentially the same as the pressure exerted on the tracheal wall, with the caveat that the cuff must be large enough to easily cover the trachea [6]. Therefore, as long as the ETT is sized appropriately, one may study the pressure acting on the tracheal mucosa by using intracuff pressures as a surrogate. ETT cuff pressure is generally measured via a purpose‐made ETT cuff manometer or an electrical transducer used for arterial pressure measurement [7]. Using clinical endpoints (e.g., absence of air leak) as guidance for cuff inflation commonly leads to hyperinflation and should therefore be avoided [8].

Cuff compliance (volume change for a given pressure change) is important to understand when modeling and testing ETT cuffs. When considering pediatric patients, a helpful concept is specific compliance, which normalizes compliance for the size of the ETT. This is calculated by dividing compliance by the residual volume [9]. Studies may test and report pressures in unrestricted cuffs, that is, open to air, or within tracheae (both simulated and real). Modern pediatric ETT cuff compliance *appears* markedly decreased—roughly fivefold—when tested inside a simulated rigid trachea compared to unrestricted simulations [6]. In this situation, the cuff is not changing per se; rather, it is impacted by the surrounding trachea. While tracheal mucosa is more easily damaged via ischemia in children due to decreased perfusion pressure, safe cuff pressures have only been determined robustly in adults [10, 11].

Varying use of cuff lubrication in both laboratory and clinical research further complicates literature evaluation [12, 13]. Lubrication does not alter cuff pressure directly yet makes a significant difference in the pressure needed to form an adequate seal in rigid tracheal models and, therefore, may be used in benchtop leakage studies [14]. It also reduces pulmonary aspiration in intubated humans by reducing fluid movement down folds or channels that are almost invariably present in any high‐volume, low‐pressure cuff but absent in low‐volume, high‐pressure cuffs [15]. Therefore, lubrication may reduce the cuff pressure necessary to prevent pneumonia [16]. The effect of lubrication on cuff‐mediated shear stress on the trachea mucosa (parallel to the plane of the tracheal wall) has not been detailed and is an area for future study. If shear stress from repeated cuff expansions and contractions is clinically relevant, it would provide support for liquid cuff inflation in air transportation, as reduced cuff adjustments would be expected. Cuff material and thickness may also play a role in the prevention of peri‐cuff leakage [14].

## 3 Cuff Pressure at Altitude

At increasing altitude, the cuff's natural expansion due to decreasing environmental pressure is restricted by the trachea, which elevates intracuff pressure. By the 1970s, it was recognized via canine surrogates that cuff pressure rises dramatically when patients are brought to high altitude [17]. Cuff pressure increases approximately linearly with altitude when studied in vitro, with greater variation found in the limited human studies that have been carried out [18, 19]. Early ex vivo and canine studies led to frequent use of liquid cuff inflation by retrievalists before any human data was available [17, 18]. At the time, pediatric tubes were generally not cuffed, and pediatric research in this area thus continued to be nonexistent. The first in vivo human study of cuff pressure change at altitude was in 2004 in South Australia. This adult‐only study found that pressure in an air‐filled cuff increased from 22 to 45.4 cm H_2_O, on average, with a 3000 ft altitude gain in a helicopter [20]. The project was stopped after an interim analysis of just 10 patients due to the clinically significant result, as it was considered unethical to continue. Two helicopter retrieval studies involving air‐filled cuffs (and mostly adults) found similar results [19, 21].

The first pediatric‐focused cuff pressure study was not performed until 2011, and it involved driving ETTs up a dormant volcano to 2400 m above sea level in Hawaii [7]. Importantly, the temperature was kept relatively constant within the car. 6.0 mm ETTs placed within a model trachea increased in pressure from 27 to 179 cm H_2_O, increasing linearly with altitude. No pressure change was found when the ETT cuff was filled with water. A more definitive study result came from a pediatric mannequin study in 2016. Relatively rigid models were intubated with appropriately sized ETTs (3.0–6.0 mm) and taken to altitude in helicopters and airplanes [22]. Starting from a cuff pressure of 10 cm H_2_O, most cuffs breached 30 cm H_2_O by 1500 ft and 50 cm H_2_O by 2800 ft.

In 2018, the first cuff‐related, in vivo study of children undergoing air retrieval was reported [23]. It was observational, describing for the first time the cuff pressure increases that occur in flown children. Pressure increased by between 23 cm H_2_O (altitude gain of 1000 ft) and 102 cm H_2_O (altitude gain of 4000 ft), assuming a starting altitude of approximately sea level. There is no data on clinical outcomes with respect to cuff pressures in pediatric retrievals.

While in vitro studies have shown a clear and linear cuff pressure increase with altitude for adults and children, the literature gives a somewhat erratic correlation between altitude and cuff pressure change in clinical studies, with significant variation between individuals [19, 21, 24]. This is poorly understood and likely multifactorial. Therefore, predicting the cuff pressure change for a given altitude in patients with air‐inflated cuffs is difficult, and a cuff manometer is necessary.

Laryngeal mask airway (LMA) cuff pressure similarly increases with altitude when filled with air [7, 25]. LMAs are sometimes used in air transport when a child cannot be intubated [26]. In this situation, a different consideration becomes important with altitude gain as when LMA cuffs are insufflated with air to an appropriate degree, a further increase in cuff pressure usually *worsens* the seal. This was first noted in adults [27] but subsequently confirmed in children [8, 28, 29, 30, 31]. Liquid LMA insufflation has been used for decades, for example, to reduce risk during laser surgery or to maintain cuff pressure stability with nitrous oxide use, while in vitro work has found liquid insufflation dampens the pressure increase seen with altitude [32, 33]. Thus, saline or water‐filled LMA cuffs are a valid consideration in air transport of children. To our knowledge, this technique has not yet been described.

Table 1 shows the variety of protocols that a retrieval team may use to manage ETT cuff pressure variation with altitude. The strategies that employ intermittent pressure assessment due to air use result in a variable period with suboptimal cuff pressure. Usually, this also means that cuffs will need air removed upon ascent and refilled upon descent. Also, upon reaching a given cruising altitude, the cuff pressure will likely decrease spontaneously over the following 10 min, perhaps reflecting a difference between the cuff's dynamic and static compliance [17]. Therefore, when using air‐filled cuffs, it is likely best practice to check the cuff pressure often throughout the flight.

**TABLE 1:** Various ETT cuff inflation recommendations have been given for aeromedical retrieval but none specifically for children.

ETT cuff management strategy	Advantages/disadvantages
Saline or water preferable [34]	Recognizes the minimal cuff pressure variation if a non‐compressible liquid is used but provides flexibility
Use saline [19]	Gives standardization but requires changing all cuffs to saline to meet protocol
Reduce cuff air by a given volume per thousand feet of ascent and return the same amount upon descent [35]	Uses an understanding of physiology. Does not take into account the variability in pressure changes seen between patients
Clinician discretion with regards to the type of cuff fluid chosen, but frequently monitor cuff pressures if air is used instead of liquid [36, 37]	Gives flexibility and recognizes significant pressure variability is seen if air is utilized. Does not specify frequency or key moments to check cuff pressure
Use air for all and check cuff pressure at certain key moments, for example, before takeoff, after ascent, and after descent [21]	Recognizes events that are likely to cause large pressure changes. Relies on the clinician to remember the task when competing interests may distract attention

## 4 The Hyperbaric Analogy

ETT cuff management in aeromedical (hypobaric) retrieval is inversely analogous to the scenario of hyperbaric therapy. This field of medicine has used liquid‐filled cuffs as a matter of course for decades, including pediatric patients [38, 39]. An intubated child generally has the ETT cuff exchanged for liquid for the hyperbaric session, and this is replaced with air when returning to the intensive care unit. However, outcome data driving this practice appear to be missing. Clinical practice in this specialty thus responded to cuff pressure measurements and a priori principles. As with the aeromedical literature, hyperbaric research has shown there can still be significant cuff pressure change using liquid‐filled cuffs. A recent study by Benzidi et al. elegantly demonstrated that this is mainly due to residual air in the cuff (as opposed to air channels outside the cuff or some other factor), with repeated air purging essentially ablating the cuff pressure variation [39]. It would logically follow that this would be the case for hypobaric scenarios as well. There is unfortunately little evidence to guide the use of liquid cuff insufflation in air transportation, and hyperbaric medicine offers little recourse other than clinical experience.

## 5 Temperature Effects

The tracheal mucosa can be used to monitor patient core temperature accurately in adults and children by using a thermocouple embedded in the cuff [40, 41]. If filled with ambient air, the ETT cuff temperature would initially be the same as the surrounding environment. It then approaches the patient's temperature over time in an ill‐defined manner. Since the tracheal mucosa's temperature is close to core temperature and the air in the ETT is warmed by the patient, the intracuff temperature would be expected to be close to that of the patient; however, this has never been studied. Notably, field temperatures might be much colder than that of the patient.

Air may be treated as an ideal gas at clinically relevant temperatures and pressures. According to the ideal gas law, the temperature of cuff air dramatically affects its pressure. Cuff pressure change (if the volume is not allowed to vary) is linearly proportional to the absolute temperature change of the cuff air. Measurements are not needed to understand the magnitude of the consequences. For example, a cuff at 20 cm H_2_O gauge pressure (1053 cm H_2_O absolute pressure) at 5°C will increase to 141 cm H_2_O gauge pressure as its temperature climbs to 37°C. Evidence of this is seen in patients undergoing induced hypothermia on cardiopulmonary bypass, who experience a linear decrease in cuff pressure as cooling progresses. This was noted first in adult cardiac operations and, 20 years later, in children [42, 43]. The temperature effect may explain some variation in results regarding cuff pressures in air transport studies [18]. Saline and water do not appreciably expand or contract with temperature variation, so temperature effects on liquid‐filled cuffs would be minimal.

## 6 Ultrasound Implications

Liquid‐filled ETT cuffs provide another advantage in retrieval scenarios: the enhanced ability to detect ETT location via transcervical ultrasonography. X‐ray and bronchoscopy are generally unavailable during retrieval; transport vehicles can be incredibly noisy (preventing accurate lung auscultation), and laryngoscopy is usually difficult due to poor patient access. Cuff palpation in the jugular notch while observing pilot balloon distension can be used as a rough indicator of position. However, ultrasound machines are now routinely carried on many air ambulances—both fixed and rotary wing—as the market has made them smaller, lighter, and less expensive. Liquid makes the ETT cuff easier to visualize than air.

First described in 1987 in adults, the use of ultrasound to identify ETT location remained an extremely rare technique for any age group until recently [44]. One study of 42 children (aged 3 months to 18 years) found about 99% sensitivity and 96% specificity with respect to tracheal intubation at a correct depth [45]. The mean scanning duration was 4 s. A more recent study of 75 children (aged 2 months to 18 years) found suprasternal visualization of a saline‐filled cuff gave an appropriate ETT depth with an accuracy of 95% [46]. All of the cuff should be placed below the cricoid cartilage, an inflexible and narrow part of the subglottic airway in children. Ultrasound can also aid in identifying the proximal shoulder of the cuff in relation to the cricoid cartilage [47].

## 7 Troubles With Liquid Cuff Insufflation

There are downsides to inflating cuffs with water or saline. This includes the time needed to evacuate the air already in the cuff and the difficulty of achieving 100% air evacuation [6, 17]. It is also possible in very austere environments that water, saline, or another liquid such as lidocaine is not available or is frozen. There have been no reports of equipment damage or failure from inflating cuffs with liquids instead of air. Services that use saline or water for aeromedical or hyperbaric purposes generally exchange this for air afterward; whether or not liquid can remain long‐term is unknown. There is some residual liquid when the cuff medium is changed to air. However, no long‐term problems have been described whether for hyperbaric or hypobaric purposes. It is thus theoretically possible to have an issue with the ETT that leads to the need for its exchange. The most likely scenario is cuff rupture due to inappropriate pressures being introduced.

There can be complications when exchanging the type of fluid for an in situ ETT, as with any clinical intervention. The most worrisome is aspiration. Extubation has never been described but is theoretically possible. Therefore, conditions should be optimized before the procedure. All necessary equipment should be confirmed present. The patient should be in a location and position on the bed that would allow reintubation. As cuff manipulation is stimulating, the patient should be deeply anesthetized and consideration given to paralysis (which is often done for air transportation anyway). The ETT securement should not be undone as it is not necessary and acts to prevent tube movement. Thorough, deep suctioning should be done immediately before deflating the cuff. If the ETT or tracheostomy tube has a subglottic suction port, this should also be suctioned, although this feature is currently only available in sizes for larger children. Performing laryngoscopy is not routinely needed as the tube itself is not replaced. However, the airway history should be reviewed so that any prior difficulties are known. Using a syringe, repeated aspiration of air bubbles in the instilled liquid may be needed to adequately remove the air. Depending on the length and diameter of the inflation line, several seconds are often needed to allow for liquid movement. Once the new fluid medium is placed, the cuff pressure must be confirmed to be in an optimal range. If the fluid is to be exchanged again after transport, this should be completed by the transporting team, and this history should be passed on to the receiving party. As with intubation, a checklist should be used to reduce errors. If the transporting clinician judges cuff medium exchange risks to outweigh the benefits, other methods to ensure an appropriate cuff pressure is maintained during the flight should be carried out.

There is a chance of cuff rupture during transport, that is, while it is filled with liquid. In the case of ETTs, the volume is sufficiently small that it should not present a risk of an aspiration syndrome. The consequences of the same situation with LMAs, which have a much larger cuff volume, are unknown. However, the bigger risk in these circumstances is likely related to the translocation of bacteria‐laden secretions into the lungs that can occur with cuff deflation, rather than the translocation of water or saline. From this perspective, the content of the cuff is less important. Table 2 summarizes tips for liquid‐filled cuffs.

**TABLE 2:** Tips on liquid‐filled airway device cuffs in air transport.

**FIGURE 1:**
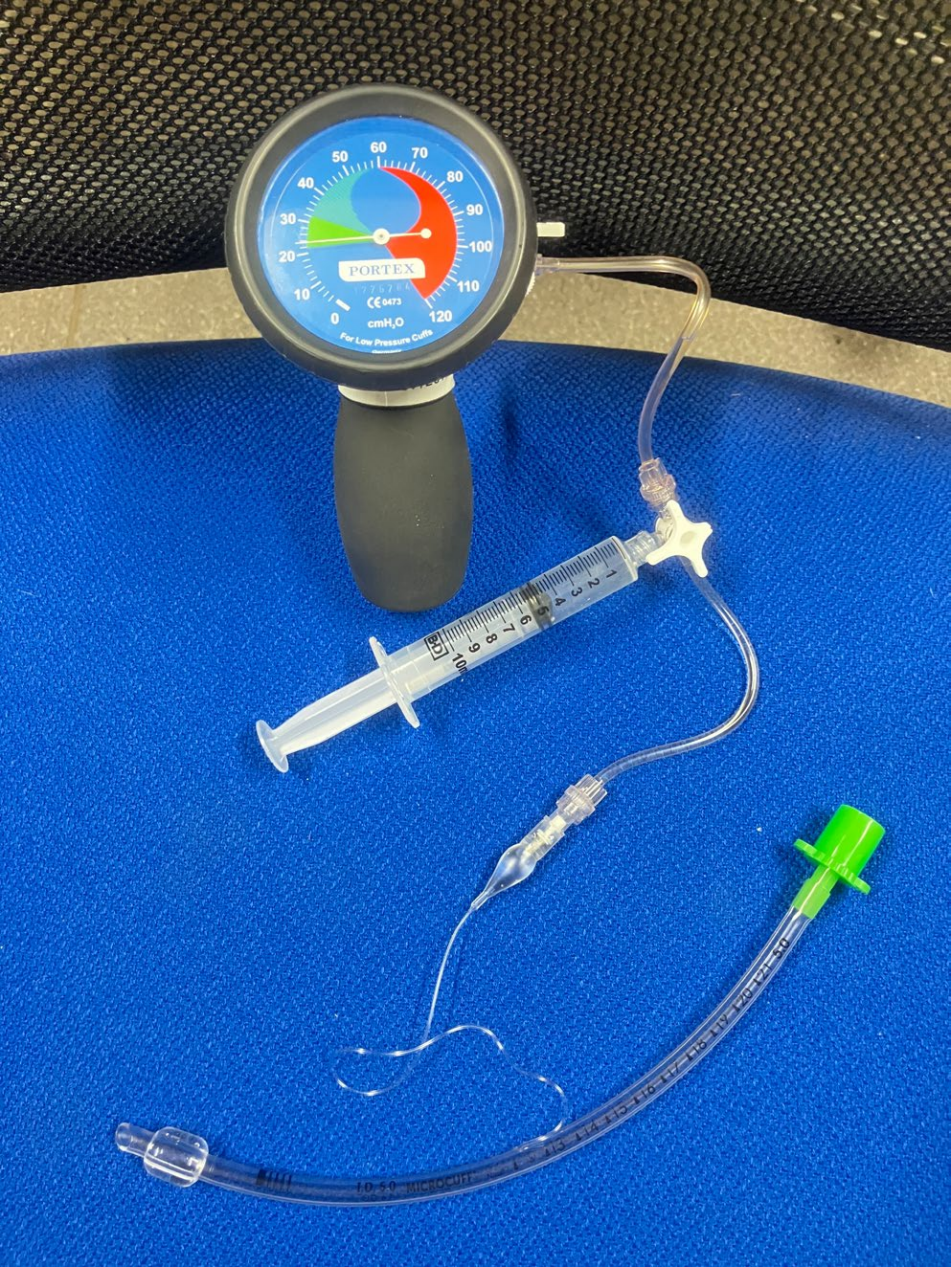
A demonstration of a setup for insufflating the cuff with liquid that provides real‐time pressure information.

Tips on liquid‐filled airway device cuffs in air transport
1. Use a checklist. This may be integrated into an extant intubation checklist, for example, or be separate
2. Evaluate the need for using liquid cuff insufflation and personal expertise, including any organizational protocols
3. Communicate the plan with the rest of the team
4. If the child is not yet intubated, replace residual air in the cuff with liquid using a syringe and repeated aspiration. (This may be done early, e.g., en route to the scene.) This avoids a common error of simply filling the cuff with liquid, leaving bubbles mixed with the liquid
5. If the child is already intubated, use the checklist to prepare for exchanging the cuff medium
6. If already intubated, the patient should be positioned in such a way—and other preparations made—to allow reintubation in the unlikely case it is needed
7. If already intubated, the child should be adequately anesthetized, consideration given to paralysis, and deep suction performed
8. When removing cuff air and replacing with liquid, a four‐way stopcock (with its three ports connected to the manometer, the syringe, and the ETT pilot balloon) can be used to allow real‐time cuff pressure observation as liquid is injected into the cuff, although there is often a time lag of several seconds (see Figure 1)
9. It is an error to not give several seconds for liquid to move and equilibrate in the inflation line and cuff, as this is much more viscous than air. Confirm cuff pressure with a manometer after sufficient time has been allotted
10. After transport, do not forget to repeat the process to replace the cuff with air
11. Communicate cuff management with the receiving team
12. Document the care

The compressible nature of air means that air‐filled cuffs have increased compliance relative to saline‐filled ones, a phenomenon also shown to occur in LMAs [29]. This can result in different pressures for the same inflation volume of liquid as air. These pressure differences are mostly notable at dangerously high cuff pressures when cuff air becomes significantly compressed and affords a protective effect. Nevertheless, this phenomenon has led to one source recommending against saline‐filled cuffs [48]. It should be noted that this recommendation comes from unpublished data derived from a laryngeal model. The protective effect of air compression relative to an incompressible liquid in an overinflation scenario is a topic for further study.

## 8 Conclusions

Altitude changes significantly alter ETT cuff pressure when insufflated with air. The dynamics are poorly studied in children and, consequently, most knowledge and practices are derived from adult studies and expert opinion. Either liquid or air can be effectively used for ETT cuff inflation. However, using an incompressible liquid such as saline or water should be strongly considered in children, given the increased consequences of tracheal trauma.

Liquid cuff inflation is likely safer from a human factor's perspective. For example, this method removes the need for the clinician to check and adjust the air pressure in the cuff repeatedly. As another illustration, a doctor may request a “sea level” flight for various reasons (e.g., hypoxemia, eye injury, or bowel obstruction) and fill the cuff with air on this assumption. If the pilot instead flies with the cabin set at higher altitudes, cuff pressure will be significantly increased without the doctor realizing it. Even with the use of protocolized or multiple cuff checks (e.g., after ascent to cruising altitude), clinicians may not be able to move out of their seats due to weather, and tracheal injury may result given the rapidity of onset. For this reason, liquid cuff inflation should be considered for even short flights. Finally, in the extremely rare event of cabin depressurization, cuff pressure would become tremendous at altitude. In this dramatic situation, the clinician is unlikely to immediately think of adjusting the cuff or even be able to do so.

Manometric monitoring of cuff pressure is beneficial whether air or liquid is used, and liquid insufflation should be considered when a manometer is not available in‐flight. Liquid insufflation is particularly advantageous when multiple altitude changes are encountered, such as in alpine environments. A knowledge of cuff pressure‐volume relationships in pediatric tubes is lacking, and this would be helpful in aeromedical retrievals and other austere environments where a manometer may not be present.

Automatic cuff pressure monitoring equipment is promising [49], especially designs that do not need to be electrically powered [50], yet are unlikely to be adopted widely in the retrieval space due to weight and space considerations until smaller devices are developed. It also has its own vagaries [51] and would need specific testing for robustness in the harsher and less controlled aeromedical setting. However, future use in certain situations is likely, such as secondary, long distance pediatric transport on large machines in which weight is not critical.

Clinical outcome studies in children would give credence to the optimal cuff management technique but would likely require very large numbers of intubated pediatric patients. Furthermore, given that the literature is clear that cuff pressure rises dramatically in air‐filled cuffs at altitude and that even short periods of high cuff pressure cause injury, such studies could be considered unethical. Indeed, one adult study was stopped early due to this conclusion [20]. Outcome studies randomizing children undergoing hyperbaric therapy to air or liquid‐filled cuffs have never been done and are unlikely ever to be undertaken; similarly, this type of study might never be carried out in pediatric air transportation.

## Conflicts of Interest

BSvUS is a section editor for Pediatric Anesthesia.

## Data Availability

The authors have nothing to report.
